# Unveiling the Emergence and Genetic Diversity of OXA-48-like Carbapenemase Variants in *Shewanella xiamenensis*

**DOI:** 10.3390/microorganisms11051325

**Published:** 2023-05-18

**Authors:** Xueqi Jiang, Beibei Miao, Xiaofei Zhao, Xuemei Bai, Min Yuan, Xia Chen, Xinyi Gong, Zeliang Liu, Jie Li, Shuang Meng, Xiao Han, Juan Li

**Affiliations:** State Key Laboratory of Infectious Disease Prevention and Control, National Institute for Communicable Disease Control and Prevention, Chinese Center for Disease Control and Prevention, Beijing 102206, China; jiangxueqi627@163.com (X.J.);

**Keywords:** *S. xiamenensis*, oxacillinases, carbapenem hydrolysis activity, OXA-48-like

## Abstract

An increase in the carbapenem-hydrolyzing capacity of class D *β*-lactamase has been observed in strains of multiple species, posing a significant challenge to the control of antibiotic resistance. In this study, we aimed to investigate the genetic diversity and phylogenetic characteristics of new *bla*_OXA-48_-like variants derived from *Shewanella xiamenensis*. Three ertapenem-non-susceptible *S. xiamenensis* strains were identified, one isolated from the blood sample of an inpatient, the other two isolated from the aquatic environment. Phenotypic characterization confirmed that the strains were carbapenemase producers and exhibited antimicrobial resistance patterns to ertapenem, with some showing lower susceptibility to imipenem, chloramphenicol, ciprofloxacin, and tetracycline. No significant resistance to cephalosporins was observed. Sequence analysis revealed that one strain harbored *bla*_OXA-181_ and the other two strains harbored *bla*_OXA-48_-like genes, with open reading frame (ORF) similarities with *bla*_OXA-48_ ranging from 98.49% to 99.62%. The two novel *bla*_OXA-48_-like genes, named *bla*_OXA-1038_ and *bla*_OXA-1039_, respectively, were cloned and expressed in *E. coli*. The three OXA-48-like enzymes demonstrated significant hydrolysis activity against meropenem, and the classical *β*-lactamase inhibitor had no significant inhibitory effect. In conclusion, this study demonstrated the diversity of the *bla*_OXA_ gene and highlighted the emergence of novel OXA carbapenemases in *S. xiamenensis*. Further attention to *S. xiamenensis* and OXA carbapenemases is recommended for the effective prevention and control of antibiotic-resistant bacteria.

## 1. Introduction

The escalation of antimicrobial resistance is an evolutionary response to the selective pressure imposed by antimicrobial agents, and the surge of carbapenem resistance genes in *Enterobacterales* has become an urgent concern [1,2]. According to the Ambler classification system, clinical β-lactamases are commonly classified into four groups (A, B, C, and D), with oxacillinases (OXA) being categorized as class D-type serine β-lactamases [3,4]. To date, there are 1237 identified class D β-lactamases [5]. The substrate spectrum of carbapenem-hydrolyzing OXA subfamilies is broadened by amino acid motif substitutions [6]. The OXA-48 family is one of the most notorious subgroups of carbapenemase-producing enzymes, presenting a significant threat to public health. Since the discovery of the first *bla*_OXA-48_ gene in *Klebsiella pneumoniae* in Istanbul in 2001, there has been a growing number of carbapenem-hydrolyzing class D β-lactamases (CHDLs) found globally [7,8,9,10,11]. Furthermore, an increasing number of OXA-48-like variants capable of carbapenem hydrolysis have been identified in both the clinical and community settings [12,12,13,14,15,16]. As carbapenems are considered last-resort antimicrobials in the treatment of Gram-negative bacterial infections, the emergence and spread of OXA variants has garnered significant attention because of the potential risks they present in clinical infection management.

The class D β-lactamases of *Shewanella* spp., encoded by chromosomes, are considered the progenitors of carbapenem-hydrolyzing OXA enzymes, such as *bla*_OXA-48_-like and *bla*_OXA-55_-like enzymes. *Shewanella* spp. are widely distributed in marine environments, including extreme ecosystems. Reports indicate that an increasing number of *bla*_OXA_-harboring *Shewanella* spp. strains are closely associated with human diseases [7,8,9,10]. The common pathogenic species of *Shewanella*, including *Shewanella xiamenensis*, *Shewanella algae*, and *Shewanella putrefaciens* [11,12], are derived from various environments such as saltmarsh plants, freshwater, estuarine water, sewage, river water and livestock wastewater [12,13,14,15,16,17]. According to the latest clinical reports, skin and soft tissue infections, intestinal colonization, respiratory diseases, bacteremia, intra-abdominal infection, and otitis media are the common complications caused by *Shewanella* spp. pathogens [18,19,20,21,22]. Although cases of clinical infection caused by *Shewanella* spp. have been reported in many countries [23,24,25], the actual incidence of these infections in China remains unclear. *S. xiamenensis* was discovered in coastal sea sediment in Xiamen, China, in 2010 [26]. To date, 26 OXAs have been reported in *Shewanella* spp. strains according to the latest β-lactamase database [5]. *Shewanella* spp. have been considered important carriers and reservoirs of carbapenem-resistant genes in the natural environment since the first report of chromosome-encoded OXA of *Shewanella oneidensis* in 2004 [27]. Additionally, there is a high risk of horizontal transfer of the chromosome-based *bla*_OXA_ variants to other *Enterobacterales* by various mobile genetic elements, such as plasmids and transposons, which dramatically reduce susceptibility to *β*-lactam antibiotics [28,29]. In this article, we investigated three carbapenem-resistant *S. xiamenensis* strains that harbored *bla*_OXA_ genes, including *bla*_OXA-181_ and two novel *bla*_OXA-48_-like genes, namely *bla*_OXA-1038_ and *bla*_OXA-1039_. This study aimed to characterize the carbapenem-hydrolyzing activity of the two new members of OXA carbapenemase and contribute to the known diversity of the OXA-type class D *β*-lactamase family identified in *Shewanella* spp. strains.

## 2. Materials and Methods

### 2.1. Strain Collection and Identification

Three carbapenem-non-susceptible strains of *S. xiamenensis* were isolated from aquatic environments (*n* = 2) and a clinical patient (*n* = 1) in China between 2019 and 2020. Species identification of the strains was confirmed by culture morphology and the VITEK 2 compact system (bioMérieux, Craponne, France). To ensure consistency, all isolates were sub-cultured at least twice prior to further investigation.

### 2.2. Antimicrobial Susceptibility Testing

The resistance profiles of *S. xiamenensis* strains were determined with the BD Phoenix™ M50 system (Becton, Dickinson and Company, Franklin Lakes, NJ, USA); the following 13 agents were included: ampicillin, ampicillin-sulbactam, aztreonam, cefotaxime, cefoxitin, ceftazidime, ceftazidime-avibactam, chloramphenicol, ciprofloxacin, ertapenem, imipenem, meropenem, and tetracycline. The minimal inhibitory concentrations (MICs) of 9 *β*-lactam antibiotics of cloned strains were obtained via broth microdilution methods.

### 2.3. Phenotypic Profiles of Isolates

Phenotypic screening for carbapenem non-susceptible strains was performed using the Carba NP test [30] and modified carbapenem inactivation method (mCIM) assay [31], with *Escherichia coli* ATCC 25922 serving as the quality control strain. All tests were performed and interpreted according to the Clinical and Laboratory Standards Institute (CLSI) M100 guidelines for *Enterobacterales* (2021).

Matrix-assisted laser desorption/ionization time-of-flight mass spectrometry (MALDI-TOF MS) (Bruker Daltonics GmbH, Bremen, Germany) was used to analyze the hydrolysis spectrum of carbapenemase-carrying *E. coli* cloning strains [32]. The positive cloned strains were induced at 0.5 mM IPTG concentration at 35 ± 2 °C for 3~4 h, and the centrifuged bacteria were incubated with meropenem solutions. A positive result for carbapenemase production was determined if the peak for meropenem at 384.5 *m*/*z* and 406.5 *m*/*z* disappeared or declined significantly, while the peak at 358.5 *m*/*z* appeared during the incubation time.

### 2.4. Whole-Genome Sequencing and Phylogenetic Analyses

The genomic DNA was extracted following the protocol of the Genomic DNA Purification Kit (Promega, Madison, WI, USA). Sequencing was performed using Illumina Miniseq and PacBio platforms. The probable antibiotic resistance genes of each sequence were detected with ResFinder 4.1 in the Center for Genomic Epidemiology database (https://cge.food.dtu.dk/services/ResFinder/) [accessed on 1 February 2023]. A phylogenetic tree of *bla*_OXA-48_-like genes of *Shewanella* spp. was constructed using MEGA 7. Based on the information, linear comparisons of sequences were made with BLAST (https://blast.ncbi.nlm.nih.gov/Blast.cgi) [accessed on 1 February 2023] and retrieved genes were aligned with the online multiple sequence alignment tool Clustal Omega (https://www.ebi.ac.uk/Tools/msa/clustalo/) [accessed on 5 February 2023]. The hypothetic draft genome sequences were annotated using the RAST server (https://rast.nmpdr.org/) [accessed on 5 February 2023].

### 2.5. Cloning and Functional Verification of Bla_OXA_ Genes

The genomic DNA of the *S. xiamenensis* strains was extracted as previously described. Specific primers were used to amplify the DNA fragments via polymerase chain reaction (PCR) with 2×*EasyTaq*^®^ PCR SuperMix (+dye). The PCR products were double-digested with *Nde* I and *Xho*l I and ligated into a pET30a (+) vector using T4 DNA Ligase (Takara Biomedical Technology, Co., Ltd., Beijing, China). The resulting ligation products were transformed into chemically competent *E. coli* DH5*α* cells via heat shock. The recombinant plasmids were then introduced into *E. coli* BL21 (DE3) chemically competent cells, which were selected on Luria–Bertani (LB) agar plates containing 50 μg/mL of kanamycin to incubate the specific *bla*_OXA_ genes. The identity of the inserts was confirmed via sequencing to ensure the integrity of encoding genes.

### 2.6. β–Lactamase Activity Assay

The OXA enzymes extracted from successfully cloned carbapenemase-producing strains were purified using the His·Bind Purification Kit (Millipore, Burlington, MA, USA). Meropenem hydrolysis activity was directly detected using the BioTek Synergy NEO system, which measures the change in absorption light (Abs) at the wavelength indicated for meropenem (310 nm). Hydrolysis rates were calculated by comparing the change in optical density (OD) using Microsoft Excel. Additionally, an in vitro inhibitory test was performed to demonstrate the hydrolysis ability of the new OXA-type carbapenemase.

## 3. Results

### 3.1. Species Isolation and Identification

Three strains of *S. xiamenensis* were identified using the VITEK 2 compact system. Among them, strain A5468 was isolated from the blood of an inpatient in Beijing, while CW86-2 and CW86-3 were derived from the aquatic environment of Lake Taihu, Jiangsu Province.

### 3.2. Antimicrobial Profile of Isolates

The broth microdilution test was used to determine the MICs of the *S. xiamenensis* strains, and the results are presented in Table 1 to display their resistance profiles. The MIC results demonstrated that all three strains were susceptible to expanded-spectrum cephalosporins, resistant to ertapenem (with MICs ranging from 2 to 4 μg/mL) and ciprofloxacin (with MIC >2 μg/mL), and showed reduced susceptibility to carbapenems, especially imipenem (with MICs ranging from 1 to 2 μg/mL). Moreover, strain A5468 showed an intermediary result to chloramphenicol (16 μg/mL), while strain CW86-3 exhibited a high level of resistance to tetracycline (with MIC >16 μg/mL).

### 3.3. Phenotypic Detection of Carbapenemase-Producing S. xiamenensis

**Figure 1:**
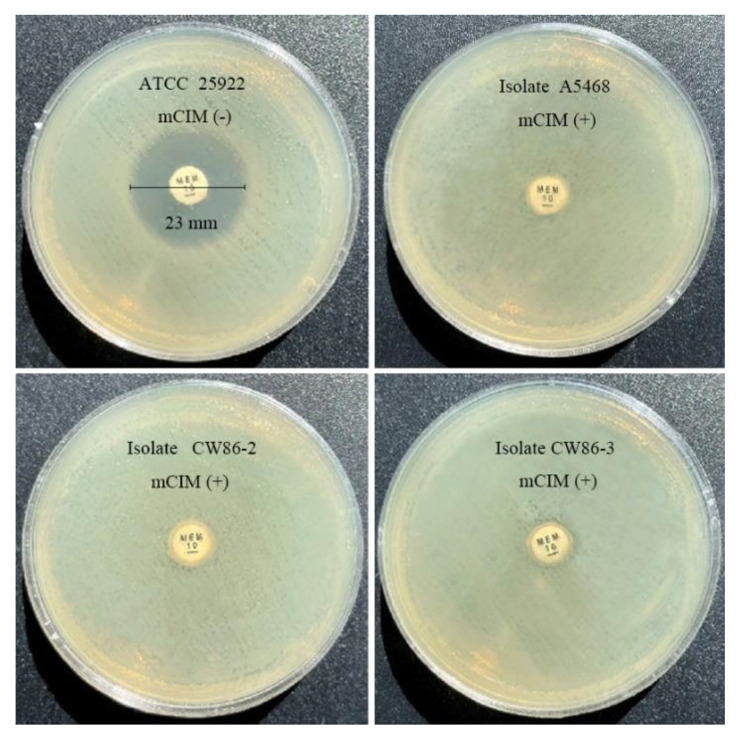
Identification of carbapenemase-producing strains via mCIM test. The control strain *E. coli* ATCC 25922 was carbapenemase negative, having a zone size of 23 mm > 19 mm. Three target isolates carried carbapenemase, which can break down 10 μg meropenem after incubation for 4 h ± 15 min at 35 °C.

The molecular phenotypic analysis employed the Carba NP test and mCIM assay for the detection of carbapenemase-producing strains, and all instructions and interpretations followed CLSI guidelines. The mCIM results of the three *S. xiamenensis* isolates are shown in Figure 1. The specificity of mCIM for detecting carbapenemase activity in the three isolates showed that they have significant carbapenemase-hydrolyzing activity compared with the negative control *E. coli* ATCC 25922.

### 3.4. Phylogenetic Analysis and Genetic Characterization of Strains

Strains A5468 and CW86-2 were subjected to sequencing with the PacBio platform, while isolate CW86-3 was sequenced with the Illumina Miniseq(MIGIGENE Company, Beijing China,). Sequence annotation showed that these strains carried *bla*_OXA-48_-like genes, and all three *bla*_OXA_ variants were located on chromosomes, as determined by comparison with conserved motifs of genes in *Shewanella* spp. Antibiotic resistance gene (ARG) screening using CGE ResFinder 4.1 revealed that strain A5468 carried *β*-lactam, sulfamethoxazole, chloramphenicol, and trimethoprim resistance genes, namely, *bla*_OXA-181_, *sul*1, *cat*A2, and *dfr*A16, respectively. Strain CW86-2 carried a novel class D *bla*_OXA-48_-like gene, while strain CW86-3 harbored another novel *bla*_OXA-48_-like gene and the tetracycline resistance gene *tet*(B). Notably, two novel *bla*_OXA-48_-like genes with >99%, but not 100%, identity to *bla*_OXA-48_ were present on their chromosomes. To confirm the presence of these two novel genes, their sequences were submitted to the NCBI BankIt database and they were designated as *bla*_OXA-1038_ (OK180617) and *bla*_OXA-1039_ (OK180618), carried by CW86-2 and CW86-3, respectively in 2021. In 2023, when the manuscript was submitted, we found that the *S. xiamenensis* strain BC01 (JGVI01000025), reported in 2014 in Xiamen, China, shares a 100% identical amino acid sequence with the OXA-1039, while differing by six nucleotides in nucleotide sequences with *bla*_OXA-1039_ mentioned in this study. Because there was no information about *bla*_OXA_ or oxacillin enzyme mentioned in the context of *S. xiamenensis* strain BC01 when it was reported in 2014 [33], on NCBI BankIt database, the oxacillin enzyme carried by the isolate CW86-3 was widely regarded as a novel oxacillin enzyme and designated as OXA-1039 in 2021. Now, the time of first report and accession number of *bla*_OXA-1039_ are can be as 2014 and JGVI01000025 in Beta-Lactamase DataBase (http://www.bldb.eu/) [accessed on 12 September 2021].

**Figure 2:**
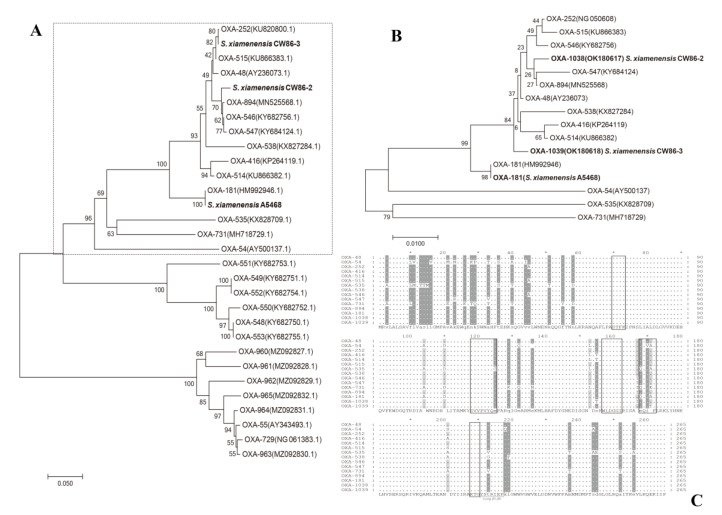
Molecular phylogeny of the representative class D OXA in *Shewanella* spp. (**A**) Phylogenetic tree with 1000 bootstrap replicates generated using the neighbor-joining (NJ) method based on the nucleotides of this study (black bold) and typical *bla*_OXA_ genes reported in *Shewanella* spp. strains. The nucleotides corresponding to the OXA-48-like cluster are outlined by the dashed box. The bootstrap values are given at branching points, and the bar represents 0.05 substitutions per nucleotide position. GenBank accession numbers are given in parentheses. (**B**) Phylogenetic analysis with an NJ tree based on the amino acids of OXAs. Comparison of 16 CDS of the *bla*_OXA-48_-like cluster mentioned in (**A**) to show the genotypic characteristics. The OXA mentioned in this study are in black bold. Bar represents 0.01 substitutions per amino acid position. Numbering is according to NCBI. (**C**) Alignment of amino acid sequences of the three OXA-type enzymes in this study with the amino acid sequences of OXA-48-like variants reported in *Shewanella* spp. from NCBI including OXA-48 (AY236073), OXA-54 (AY500137), OXA-252 (NG_050608), OXA-416 (KP264119), OXA-514 (KU866382), OXA-515 (KU866383), OXA-535 (KX828709), OXA-538 (KX827284), OXA-546 (KY682756), OXA-547 (KY684124), OXA-731 (MH718729), OXA-894 (MN525568), OXA-181 (this study), OXA-1038 (OK180617, this study) and OXA-1039 (OK180618, this study). The bottom line shows the consensus sequence of all *bla*_OXA_ genes. Dashes indicate identical residues among all the amino acid sequences. Amino acid motifs that are well-conserved among class D *β*-lactamases are indicated by black-outlined boxes, and the single gray-outlined box corresponds to the *β*5-*β*6 loop [36,37]. Differences in residues within three kinds of amino acids among all sequences are shaded in dark gray, and differences of more than three are shaded in light gray.

Based on the ORFs of all *bla*_OXA_ genes in *Shewanella* spp., the three genes mentioned in this study belong to a cluster of *bla*_OXA-48_ genes, as shown in the dashed box in Figure 2A. The phylogenetic tree of 16 amino acid coding sequences (CDS) of the OXA-48-like cluster is depicted in Figure 2B, and some of the OXA variants in this cluster were found to possess carbapenem hydrolysis activity. Phylogenetic analysis based on CDS indicated that OXA-181, OXA-1038, and OXA-1039 had high similarity with other OXA-type carbapenemases in this cluster. The 265 amino acid sequence alignment of the cluster of OXA-48 subgroup variants is shown in Figure 2C. Partial *bla*_OXA_ genes displayed amino acid motif mutations in consensus regions. Sequencing analysis revealed that *bla*_OXA-181_ has 98.49% (261/265) identity to *bla*_OXA-48_ and its genome characteristics have been previously reported [12,34,35]. The *bla*_OXA-1038_ gene of strain CW86-2 shared 99.62% and 99.25% amino acid identity with *bla*_OXA-894_ (S155T) and *bla*_OXA-48_ (S155T, T167I), respectively. The *bla*_OXA-1039_ gene shared 99.62% with *bla*_OXA-48_ (S171T), 99.25% with *bla*_OXA-894_ (I167T, S171T), and 99.25% with *bla*_OXA-252_ (A45V, S171T).

### 3.5. Hydrolytic Spectra of OXA-48-like-Carrying Cloning Strains

The 798 bp PCR target product of the three *bla*_OXA_ open reading frames (ORFs) was cloned into *E. coli* BL21 chemically competent cells after being transformed into *E. coli* DH5*α* competent cells using *E. coli* pET30a (+) plasmids as the recipient strains. The colonies were incubated and screened using X-Gal and IPTG in the 50 μg/mL kanamycin-containing LB culture medium, and the integrity of the gene fragment of each recombinant strain was confirmed using Sanger sequencing. The resistance profiles of the recombinant plasmids pET30a-OXA-181, pET30a-OXA-1038, and pET30a-OXA-1039 were compared with those of the *E. coli* reference strains for nine *β*-lactam antibiotics, as shown in Table 2. The expression efficiencies of all the OXA variants against imipenem were higher than non-OXA strains, with MICs of 1 μg/mL in *E. coli* cloning strains. Furthermore, compared with the MICs of the original *S. xiamenensis*, only the *bla*_OXA-181_-gene-harboring strain showed higher hydrolysis to ampicillin, and no cloning strain had significant intrinsic activity towards ertapenem, meropenem, and expanded-spectrum cephalosporins.

**Figure 3:**
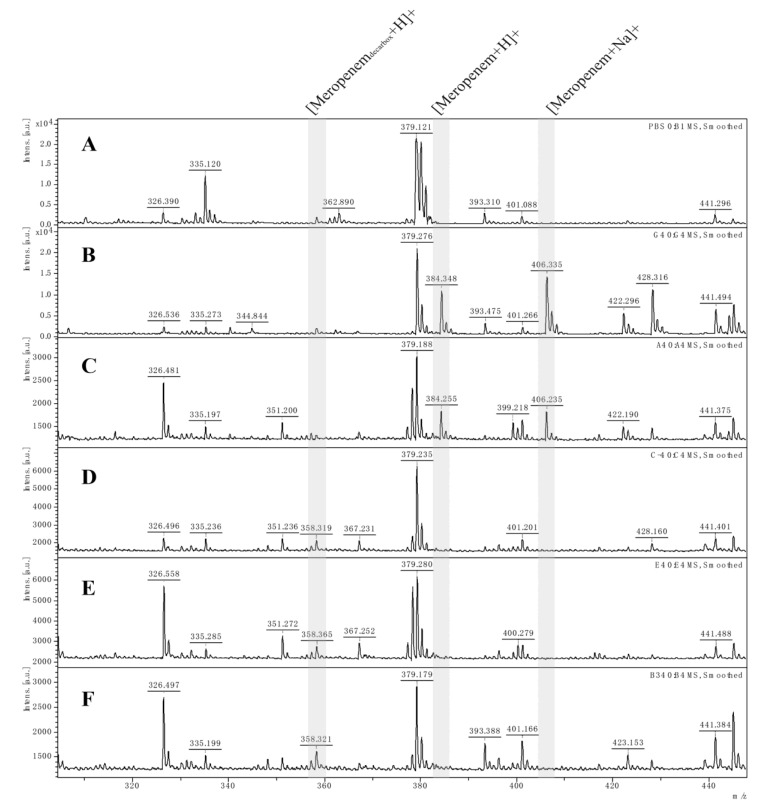
The MALDI-TOF MS spectra of 0.5 mg/mL meropenem. (**A**) The spectrum of PBS was included to rule out interference from matrix fluids. (**B**) Blank control spectrum of pure meropenem solution. (**C**) Negative control: spectrum of a carbapenemase-non-carrying strain. (**D**–**F**) Positive: OXA-181-harboring strain, OXA-1038-harboring strain and OXA-1039-harboring strain, respectively. Shaded area: [Meropenem_decarbox_ + H]^+^, decarboxylated degradation product of meropenem after carbapenemase hydrolysis (*m*/*z* 358.5); [Meropenem + H]^+^, meropenem molecule (*m*/*z* 384.5); [Meropenem + Na]^+^, meropenem sodium salt (*m*/*z* 406.5).

The MALDI-TOF MS assay was implemented to detect hydrolysis of recombinant cloning strains to five *β*-lactam antibiotics, including ampicillin, cefotaxime, ceftriaxone, meropenem and imipenem [38,39,40]. After incubation of antibiotics solution as the substrate with different strains for 4 h, the performance of three cloning strains was evaluated. Figure 3 shows the hydrolysis mass spectrum of meropenem. The cloning strains were judged as positive OXA-type carbapenemase-producing strains if the specific peak for meropenem at 384.5 *m*/*z* and its monosodium salt at 406.5 *m*/*z* disappeared or declined significantly, while its decarboxylated degradation product at 358.5 *m*/*z* appeared during the incubation time [41]. It seems that *bla*_OXA-181_-, *bla*_OXA-1038_- and *bla*_OXA-1039_-carrying *E. coli* cloning strains have a low level of carbapenemase hydrolysis activity similar to OXA-48-like variants previously reported in *Shewanella* spp. isolates [13,22,42].

### 3.6. Purification and Biochemical Properties of OXA-Type Carbapenemase

**Figure 4:**
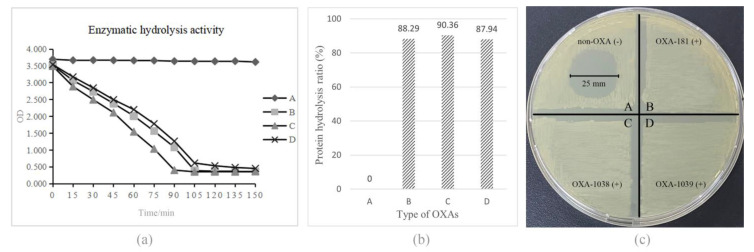
Results of the evaluation of the meropenem hydrolysis ability of the three OXA enzymes. (**a**) Verification of the hydrolysis ability of the three new enzymes (A: PBS, negative control; B: OXA-181; C: OXA-1038; D: OXA-1039). (**b**) Comparison of the hydrolysis rates of PBS, OXA-181, OXA-1038 and OXA-1039. All OXA enzymes showed a significant ability to hydrolyze meropenem. (**c**) Results of the in vitro enzyme inhibition test, which further confirmed the hydrolysis ability of the OXA enzymes. All OXA-enzyme-carrying cloning strains showed a significantly higher ability to hydrolyze meropenem compared to the non-OXA-carrying negative control (A = 25 mm).

The ability of different OXA enzymes to hydrolyze meropenem, imipenem, and classical *β*-lactam inhibitor solutions was measured via Abs readings continuously monitored at 37 °C for 2 h. The meropenem hydrolysis rates were calculated from the change in absorbances at 310 nm per unit time (Figure 4a), and no OXA enzymes were inhibited by classical enzyme inhibitors (not shown). The results from the enzyme activity test showed that all OXA enzymes had significant meropenem hydrolysis with an efficiency of more than 85% (Figure 4b). In addition, based on the carbapenemase phenotypic test, in vitro enzyme inhibition testing was carried out with antimicrobial solutions to make the hydrolysis results more visible. Meropenem hydrolysis activities were measured with purified protein extracts of the three *E. coli* cloning strains obtained as previously described, and 100 μg/mL meropenem was used as the substrate for overnight incubation at 37 °C for 2 h. A 5 μL bacterial culture suspension was added on MH agar plates, upon which a carbapenem-susceptible reporter *E. coli* ATCC 25922 had been freshly applied. The results indicated that the OXA-1038 and OXA-1039 enzymes hydrolyzed meropenem dramatically (Figure 4c). 

## 4. Discussion

Chromosome-mediated class D *β*-lactamases identified in *Shewanella* spp. were previously thought to be the progenitors of carbapenem-hydrolyzing OXA-48-like enzymes. Furthermore, it was recently discovered that an expanding number of members within the OXA-48-like subfamily possess the ability to hydrolyze *β*-lactam antibiotics, including carbapenems. *Shewanella* spp., such as *S. xiamenensis*, *S. algae*, and *S. putrefaciens*, which are commonly found in marine sediments and ecosystems, have been increasingly recognized as opportunistic pathogens of humans and animals, particularly those exposed to livestock wastewater and aquaculture environments [43,44,45,46]. Although the antibiotic resistance statuses of *Shewanella* spp. are relatively low compared to Enterobacteriaceae, *bla*_NDM_, *bla*_SHV_, *bla*_CTX-M_, *qnr*A, *cat*A, *dfr*A, and *tet* cluster genes have been reported in *Shewanella* spp. [47,48,49,50,51]. It is clear that the emergence of multiple drug-resistant *Shewanella* spp., especially those containing *bla*_OXA-48_-like genes, poses a significant threat to clinical treatment [50,52]. 

The phylogenetic analysis revealed that the OXA family could be classified into three clusters, including the OXA-48-like, OXA-55-like and OXA-548-like clusters [5]. Carbapenem-hydrolyzing activity has been reported for almost all OXA-48-like and OXA-55-like enzymes in *Shewanella* spp. but the details of the carbapenem-hydrolyzing activity of the OXA-548-like enzymes have not yet been determined. The three OXA involved in this study all belonged to the OXA-48-like cluster. The coding sequences of the three *bla*_OXA_ genes had >99% identity with *bla*_OXA-48_ and some of its variants. OXA-1038 had two amino acid differences from OXA-48 (S155T, T167I) in non-conserved regions, and OXA-1039 had only one mutation from OXA-48 in the conserved region (S171T). Partial amino acid residues of new OXA variants, such as OXA-535 and OXA-731, showed more favorable interaction between carbapenem antibiotics and residues, and we raised rational doubt that the substitution residues of OXA-1038 and OXA-1039 may also play a vital role in the evolution of carbapenemases. 

The MICs of the *bla*_OXA_ gene recombinant plasmids pOXA-181, pOXA-1038 and pOXA-1039 in BL21 (DE3) strains showed minor differences in antimicrobial susceptibility compared to the original BL21 (DE3) strains; except for a slight decrease in the sensitivity of pOXA-181 to ampicillin, the MICs of all strains to the antibiotics used did not reach the resistant level. Hydrolysis examinations showed that all recombinant strains demonstrated various sensitivity profiles against ampicillin, cefotaxime, ceftriaxone, meropenem, and imipenem, and the expression of all alleles exhibited hydrolysis activity to meropenem, with decarboxylated (*m*/*z* 358.5) and degradation (*m*/*z* 384.5, 406.6) products of meropenem degradation identified after co-incubation. All enzyme activity tests confirmed that the OXA enzyme variant OXA-181 has the same resistance profile as previously reported, while OXA-1038 and OXA-1039 possess resistance patterns comparable to OXA-181 carbapenemase. Overall, these findings indicate that all OXA enzymes in this study have a significant ability to resist meropenem, as demonstrated by their hydrolysis rates and strong in vitro enzyme inhibition test results.

The three class D-type *bla*_OXA_ genes were all found on the chromosome of *S. xiamenensis*, and the surrounding regions both upstream and downstream of these genes did not contain any mobile genetic elements, such as phages, insertion sequences, transposons, or integrons. This indicates that the transfer ability of the two novel OXA variants from *S. xiamenensis* to other species is relatively low at present. However, considering the increasing reports of *S. xiamenensis* as a clinical pathogen [28,29,30,31,32], it is necessary to strengthen the monitoring of OXA in *Shewanella* spp. strains. 

## 5. Conclusions

In this study, OXA-1038 and OXA-1039, two novel OXA variants with carbapenem hydrolysis activity, were identified in *S. xiamenensis* strains isolated from aquaculture samples. The discovery expanded the known diversity of the CHDL family identified in *Shewanella* spp. In addition, an OXA-181-carrying *S. xiamenensis* strain from a blood sample of an inpatient was detected, which increased the clinical treatment burden because of β-lactam and carbapenem resistance. Furthermore, increased attention should be given to the emergence and evolution of OXA carbapenems in order to prevent transmission between the environment, animals, and humans.

## Figures and Tables

**Table 1:** MICs of 13 antimicrobial agents for the three *S. xiamenensis* isolates.

Antimicrobial Agent	MIC (μg/mL)		
	*S. xiamenensis* A5468	*S. xiamenensis* CW86-2	*S. xiamenensis* CW86-3
Ampicillin	<=2	<=2	<=2
Ampicillin-Sulbactam	2/1	2/1	<=1/0.5
Aztreonam	2	1	0.5
Cefotaxime	1	<=0.25	<=0.25
Cefoxitin	4	<=2	<=2
Ceftazidime	4	0.5	1
Ceftazidime-Avibactam	<=0.25/4	<=0.25/4	<=0.25/4
Chloramphenicol	16	<=4	<=4
Ciprofloxacin	>2	>2	>2
Ertapenem	4	4	2
Imipenem	2	1	1
Meropenem	1	1	0.5
Tetracycline	<=1	2	>16

**Table 2:** The *β*-lactam MICs of *E. coli* harboring *bla*_OXA_ recombinant plasmid pET30a strains and BL21 (DE3) reference strain.

Isolate	MICs (μg/mL) ^a^								
	AMP	AMS	ATM	CTX	FOX	CAZ	ETM	IMP	MEM
BL21(pET30a-OXA-181)	4	2/1	1	<=0.25	<=2	<=0.25	<=0.25	1	0.5
BL21 (pET30a-OXA-1038)	<=2	2/1	<=0.25	<=0.25	<=2	<=0.25	<=0.25	1	0.5
BL21 (pET30a-OXA-1039)	<=2	<=1/0.5	<=0.25	<=0.25	<=2	<=0.25	<=0.25	1	<=0.125
BL21 (pET30a)	<=2	<=1/0.5	<=0.25	<=0.25	<=2	<=0.25	<=0.25	0.5	<=0.125
BL21 (DE3)	<=2	<=1/0.5	<=0.25	<=0.25	<=2	<=0.25	<=0.25	0.5	<=0.125

^a^ AMP, ampicillin; AMS, ampicillin-sulbactam; ATM, aztreonam; CTX, cefotaxime; FOX, cefoxitin; CAZ, ceftazidime; ETM, ertapenem; IPM, imipenem; MEM, meropenem.

## Data Availability

The two novel OXA varieties sequence OXA-1038 (https://www.ncbi.nlm.nih.gov/nuccore/OK180617), and OXA-1039 (https://www.ncbi.nlm.nih.gov/nuccore/OK180618). All the detail information have submitted to National Microbiology Data Center (NMDC) (https://nmdc.cn/submit/dashboard).
